# Organophosphorus Pesticide Exposure at 17 Weeks’ Gestation and Odds of Offspring Attention-Deficit/Hyperactivity Disorder Diagnosis in the Norwegian Mother, Father, and Child Cohort Study

**DOI:** 10.3390/ijerph192416851

**Published:** 2022-12-15

**Authors:** Amber M. Hall, Jake E. Thistle, Cherrel K. Manley, Kyle R. Roell, Amanda M. Ramos, Gro D. Villanger, Ted Reichborn-Kjennerud, Pål Zeiner, Enrique Cequier, Amrit K. Sakhi, Cathrine Thomsen, Heidi Aase, Stephanie M. Engel

**Affiliations:** 1 Department of Epidemiology, Gillings School of Public Health, University of North Carolina at Chapel Hill, Chapel Hill, NC 27599, USA; 2 Department of Child Health and Development, Division of Mental and Physical Health, Norwegian Institute of Public Health, 0213 Oslo, Norway; 3 Institute of Clinical Medicine, University of Oslo, 0315 Oslo, Norway; 4 Department of Mental Disorders, Division of Mental and Physical Health, Norwegian Institute of Public Health, 0213 Oslo, Norway; 5 Division of Mental Health and Addiction, Oslo University Hospital, 0424 Oslo, Norway; 6 Department of Food Safety, Division of Climate and Environmental Health, Norwegian Institute of Public Health, 0213 Oslo, Norway

**Keywords:** ADHD, hyperkinetic disorder, organophosphorus pesticide, prenatal exposure, MBRN, MoBa, diethylphosphate, dimethylphosphate, dietary exposure

## Abstract

Prenatal organophosphorus pesticides (OPs) are ubiquitous and have been linked to adverse neurodevelopmental outcomes. However, few studies have examined prenatal OPs in relation to diagnosed attention-deficit/hyperactivity disorder (ADHD), with only two studies exploring this relationship in a population primarily exposed through diet. In this study, we used a nested case-control study to evaluate prenatal OP exposure and ADHD diagnosis in the Norwegian Mother, Father, and Child Cohort Study (MoBa). For births that occurred between 2003 and 2008, ADHD diagnoses were obtained from linkage of MoBa participants with the Norwegian Patient Registry (N = 297), and a reference population was randomly selected from the eligible population (N = 552). Maternal urine samples were collected at 17 weeks’ gestation and molar sums of diethyl phosphates (ΣDEP) and dimethyl phosphates metabolites (ΣDMP) were calculated. Multivariable adjusted logistic regression models were used to estimate the association between prenatal OP metabolite exposure and child ADHD diagnosis. Additionally, multiplicative effect measure modification (EMM) by child sex was assessed. In most cases, mothers in the second and third tertiles of ΣDMP and ΣDEP exposure had slightly lower odds of having a child with ADHD, although confidence intervals were wide and included the null. EMM by child sex was not observed for either ΣDMP or ΣDEP. In summary, we did not find evidence that OPs at 17 weeks’ gestation increased the odds of ADHD in this nested case-control study of ADHD in MoBa, a population primarily experiencing dietary exposure.

## 1. Introduction

Attention-deficit/hyperactivity disorder (ADHD) affects 3 to 6% of children worldwide [1,2] and is diagnosed more frequently in boys compared to girls [3]. Individuals with ADHD have been shown to have lower academic [4,5] and work performance [6], less work stability [6], more difficulty with professional [6] and personal [7] relationships; and lower overall quality of life [8,9]. Heritable factors are estimated to account for 70 to 80% of ADHD cases [10]. However, there is a substantial need for identifying modifiable environmental risk factors that may be amenable to intervention. Prenatal environmental exposures are of particular interest as the prenatal period is a vulnerable time due to rapid brain growth and development [11,12,13].

One group of potential environmental risk factors for ADHD are organophosphorus pesticides (OPs). OPs are insecticides that were originally used as nerve agents during conflicts in the 1930s and 1940s, with some later used in lower doses as insecticides [14,15]. At high doses, OPs inhibit carboxyl ester hydrolases, mainly acetylcholinesterase (AChE), which results in overstimulation of nicotinic and muscarinic receptors [14]. Adverse effects of chronic low-dose OP exposure (i.e., exposure below the level expected to result in appreciable AChE inhibition) have been observed, although the mechanism of action is less established [14]. Regardless, various mechanisms have been proposed to explain low-dose effects, including changes to the methylation of DNA [16], neuroinflammation [17], interference with neural cell differentiation through signaling cascades [18], inhibition of DNA synthesis [18,19], and effects on insulin resistance [20].

Epidemiological studies have observed associations between prenatal OP exposure and adverse neurodevelopmental and behavioral outcomes in children including increased risk for autism [21,22], decreased motor function [23,24,25], increased executive dysfunction [26,27], and decreased IQ [28,29,30] in addition to several other neurodevelopmental endpoints [31,32]. To date, eight studies have examined the relationship between prenatal OP exposure and ADHD or ADHD-like symptoms, with mixed results [33,34,35,36,37,38,39,40]. In addition, two recent studies investigating impacts on offspring executive function, which are often impaired among children with ADHD, found that higher prenatal OP exposure was associated with decrements in offspring executive function across parent- and teacher-ratings as well as performance-based measures [39,41].

The bulk of the neurodevelopmental literature includes studies conducted in agricultural settings proximate to agricultural OP applications or in urban populations that were enrolled during a period where residential applications of OPs were allowed [31,32]. However, dietary exposure to OPs is likely the most common route of exposure in the general population [42], especially in countries such as Norway where the pest population is lower compared to more southern latitudes [43]. In these environments, imported food may be an important route of exposure to consider as pesticides are routinely applied to fruit and vegetable crops to prevent loss through insects [43,44,45,46].

In this study, we sought to investigate the association of prenatal OP exposure with clinical ADHD diagnosis, while accounting for the potential beneficial role of a diet rich in fruit and vegetables. We additionally explored the role of child sex as a potential modifier of this relationship, given the pronounced sex disparity in ADHD diagnosis. We hypothesized that higher OP exposure would be associated with greater odds of an offspring ADHD diagnosis, with stronger associations among boys compared to girls.

## 2. Materials & Methods

### 2.1. Study Population

We utilized a sub-study of the Norwegian Mother, Father, and Child Cohort Study (MoBa). MoBa is a prospective population-based cohort that enrolled participants between 1999 and 2008 [47]. All Norwegian speaking pregnant people were considered eligible [47]. Individuals were recruited at their first ultrasound appointment (~17 weeks’ gestation) at which time a spot urine sample was collected [47]. Over the course of MoBa, 41% of the invited 227,702 pregnancies enrolled (N = 112,908) [47]. MoBa was approved by the Regional Committee for Medical Research and the Norwegian Data Inspectorate, and written informed consent was obtained from study participants. Mothers completed questionnaires at 17-, 22-, and 30- weeks’ gestation as well as longitudinally after birth [47]. Data on pregnancy health, delivery procedures and pregnancy outcomes were obtained through linkage with the Medical Birth Registry of Norway (MBRN) [47].

Children were eligible for the present study if they were born after 2002 (N = 60,835), had completed a 36-month postnatal questionnaire (N = 34,190 remaining), did not have Down’s syndrome or cerebral palsy (N = 34,099 remaining), had available maternal biospecimens (N = 28,097 remaining), were the result of a singleton pregnancy (N = 27,347 remaining), and resided within close proximity of Oslo (the location of the clinic assessment; N = 24,035 remaining) [48]. From this final eligible population, we linked with the Norwegian Patient Registry (NPR) to identify diagnosed cases of ADHD.

### 2.2. Selection of ADHD Cases

ADHD diagnoses were obtained from linkage of study participants with NPR, as previously described by Engel (2018) et al. [48]. NPR is a medical record database from government funded facilities linked to the government reimbursement system. Reporting is mandatory and captures an estimated 90% to 95% of ADHD cases in Norway [49]. ADHD cases were coded using ICD-10 classification (ICD-10 codes: F90, F90.0, F90.1, F90.8, F90.9) [50] and 2 registrations were required to exclude false diagnoses and coding errors. A total of 298 ADHD cases met the inclusion criteria.

### 2.3. Selection of the MoBa Reference Population

We randomly selected 554 mother-child pairs from the final MoBa eligible population to represent the exposure distribution in the population of pregnancies that gave rise to the cases; we refer to these mother-child pairs as the MoBa reference population. As only two individuals that were identified as diagnosed ADHD cases via the NPR were also randomly sampled into our MoBa reference population, we decided to analyze this study as a nested case-control study. As such, these 2 overlapping individuals were treated as cases, decreasing the number of mother-child pairs in the MoBa reference population from 554 to 552.

### 2.4. Measurement of OP Metabolites

Maternal spot urine samples were collected at the mother’s first ultrasound appointment (~17 weeks’ gestation). Details regarding shipment, storage, and quality assurance and control procedures for MoBa urine samples have been detailed previously [51,52]. Briefly, urine samples were shipped unrefrigerated to a central ISO-certified lab in Oslo (Biobank) in commercially-available urine transport tubes with a preservative (chlorhexidine plus ethyl paraben and sodium propionate) to prevent bacterial growth [53]. Most samples were received within one (66%) or two (10%) days of collection [51]. Upon receipt, urine samples were processed and stored at −80 degrees C [53].

Measurement of dialkylphosphate (DAP) metabolites was conducted at the Norwegian Institute of Public Health using a ultra-performance liquid chromatography-time-of-flight system [54]. The six DAPs measured include three dimethyl phosphates [dimethyl phosphate (DMP), dimethyl thiophosphate (DMTP), and dimethyl dithiophosphate (DMDTP)] and three diethyl phosphates [diethyl phosphate (DEP), diethyl thiophosphate (DETP), and diethyl dithiophosphate (DEDTP)]. These metabolites represent a large portion of OPs, where two or more metabolites may correspond to the same parent compound [55,56].

To assess assay performance, procedural blank samples and two in-house control samples were included per analytic batch as well as 4–6 laboratory-blinded quality control (QC) pooled urine samples. Samples were randomized to analytic batch and technicians were blinded to outcome status. Specific gravity was measured using a pocket refractometer (PAL-10S) from Atago. One urine sample was excluded as an OP metabolite measurement could not be obtained; this decreased the number of ADHD cases from 298 to 297.

The percent above limit of detection (LOD) for DMP, DMTP, DMDTP, DEP, and DETP were 66.1%, 99.9%, 28.9%, 98.7%, and 66.8% respectively; values of these metabolites below the LOD were imputed from a log-normal distribution truncated at the LOD [57]. DEDTP was excluded as only 2.4% of DEDTP values were above the LOD [57]. Metabolites were then specific gravity corrected to account for urine dilution using Equation (1):

(1) $${P^{*}}_{\mathrm{ij}}\;=\;P_{\mathrm{ij}}\;\times \frac{c-1}{{\mathrm{SG}}_{j}-1}$$

where, P*_ij_ is the specific gravity corrected value of P_ij_, P_ij_ is the measured or imputed value of the OP i for participant j, SG_j_ is the specific-gravity for participant j, and c the geometric mean of specific-gravity across all participants for the OP i.

Next, metabolites were converted from ng/mL to nmol/L by dividing each metabolite by its respective molecular mass. OP metabolites were next grouped by molecular weight, thus ΣDMP was the sum of DMP, DMTP, and DMDTP and ΣDEP was the sum of DEP and DETP [58]. Calculation of ΣDMP and ΣDEP can be seen in Equations (2) and (3) respectively:

(2) $${\Sigma \mathrm{DMP}}_{j}=\frac{{\mathrm{DMP}}_{j}}{126,048}+\frac{{\mathrm{DMTP}}_{j}}{141,101}+\frac{{\mathrm{DMDTP}}_{j}}{158,170}$$

where, ΣDMP_j_ is molar sum of DMP, DMTP, and DMDTP for a participant, j. DMP_j_ is the DMP measured or imputed value for participant j. DMTP_j_ is the DMTP measured or imputed value for participant j. DMDTP_j_ is the DMDTP measured or imputed value for participant j. All values are in in nanomole/liter.

(3) $${\Sigma \mathrm{DEP}}_{j}=\frac{{\mathrm{DEP}}_{j}}{153,094}+\frac{{\mathrm{DETP}}_{j}}{169,155}$$

where, ΣDEP_j_ is molar sum of DEP and DETP for a participant j. DEP_j_ is the DEP measured or imputed value for participant j. DETP_j_ is the DETP measured or imputed value for participant j. All values are in in nanomole/liter.

Evaluation of DAPs as molar sums within their respective subgroups (ΣDMP and ΣDEP) rather than evaluating the six DAPs individually follows usual practice as multiple DAPs can result from metabolism of the same parent compound [55,56].

### 2.5. Potential Confounders

Maternal age at delivery (years), child sex at birth (male, female), and birth year (2003–2004, 2005, 2006, 2007–2008) were obtained from the MBRN. Maternal education (less than a 4-year college degree, a 4-year college degree, more than a 4-year college degree), financial difficulty experienced in the past 12 months (yes, no), marital status (single, co-inhabiting, married), parity (nulliparous, parous), maternal smoking during pregnancy (yes, no), maternal drinking during pregnancy (yes, no), maternal exposure to pesticides in the past 6 months (yes, no), paternal exposure to pesticides in the past 6 months (yes, no), residing on a farm or detached home (home not attached to another home such as in an apartment complex or condominium) during pregnancy (yes, no), and season of urine collection (fall, winter, spring, summer) were obtained from the maternal questionnaire administered at 17 weeks’ gestation. Maternal history of depression (yes/no) was also collected from this questionnaire using a dichotomized version of the lifetime history (LTH) of major depression (MD) assessment [59]. Raw fruit and vegetable consumption, frequency of organic fruit and vegetable consumption (yes/no), and total fish consumption were derived from a semiquantitative food frequency questionnaire administered at 22 weeks’ gestation. From this questionnaire, raw fruit consumption, raw vegetable consumption, and total fish consumption were estimated by converting daily, weekly, and monthly intake to servings per day for fruit and vegetable intake and grams per day for total fish intake. Maternal ADHD symptoms (yes, no) were determined using the Adult ADHD Self-Report Scales (ASRS) screener which was completed as part of the 36 months postpartum questionnaire [60].

### 2.6. Statistical Analysis

Descriptive statistics were generated for all variables. Spearman correlations between ΣDMP and ΣDEP were calculated. Missing covariate data can be found in Table 1 and was imputed using a multivariable imputation by chained equations (MICE) approach (*m* = 20). MICE is sometimes referred to as fully conditional specification, where missing values are imputed from conditional models. To apply this approach, a random value was selected from an appropriate distribution (representative of the data), conditional on the outcome, exposure, and other covariates. Summary estimates from the combined generated datasets were derived using Rubin’s rules for imputation [57,61,62,63].

We used adjusted logistic regression models to estimate associations between prenatal ΣDEP and ΣDMP exposure and offspring ADHD. Functional form assessment of ΣDMP and ΣDEP using Akaike Information Criterion identified OP tertiles as the most appropriate descriptor of OP exposure.

Potential confounders were identified from the literature and their relationships were examined using a direct acyclic graph (DAG) [64]. The minimally sufficient adjustment set based on the DAG included birth year, fish consumption, season of urine collection, family income, fruit and vegetable intake, organic food consumption, maternal age at delivery, and maternal education. Starting with this adjustment set, we eliminated covariates that had a minimal impact on estimated associations for parsimony. Final models included season, birth year, maternal education, fruit and vegetable intake, family income, maternal ADHD and child sex. Maternal ADHD was included because ADHD has substantial heritability [65,66,67,68], and maternal ADHD symptoms and may affect the potential for OP exposure through other pathways (e.g., diet). In addition, our final models simultaneously estimated associations for ΣDEP and ΣDMP in order to address the potential for confounding by pesticide exposures in the alternate class (DMP or DEP respectively).We examined multiplicative effect measure modification (EMM) of OP-ADHD associations by child sex using an augmented product term approach [69]. All analyses were conducted using version 9 of the quality-assured MoBa data files in SAS 9.4 (Cary, NC, USA).

### 2.7. Sensitivity Analysis

Two sensitivity analyses were performed. To determine the impact of imputation, primary models were rerun without imputing data (i.e., a complete case analysis). To evaluate the impact of adjusting for the other OP molar sum, models were rerun without mutually adjusting for the other metabolite class.

## 3. Results

Characteristics of the study population can be seen in Table 1. The average maternal age at delivery was around 30 years old. When compared to the MoBa reference population, children with ADHD were more likely to be male (72.3% vs. 49.6%) and to have mothers with less educational attainment (61.8% vs. 23.3% not completing a 4-year college degree), more financial difficulty (38.1% vs. 13.3%), and that reported having maternal ADHD symptoms themselves (8.0% vs. 3.0%). Each covariate had less than 10% of values missing, except paternal exposure to pesticides in the prior 6 months (19.9% missing), and maternal self-reported ADHD symptoms (20.1% missing). Missing covariate data were more extensive among children with ADHD compared to the MoBa reference population.

Specific-gravity and non-specific-gravity adjusted OP metabolite distributions are in Table 2 and Table S1, respectively. For all OP metabolites, geometric means were higher in the MoBa reference population compared to the ADHD case group. Additionally, ΣDMP and ΣDEP appeared to be moderately correlated, with Spearman correlations of 0.489 among ADHD cases and 0.524 in the MoBa reference population (Table S2).

We observed no association between ΣDMP or ΣDEP and offspring ADHD, either overall or within sex-specific strata (Table 3). Although point estimates were generally below the null, confidence intervals were wide (particularly among girls after stratification by sex). Imputation of missing data did not materially impact estimates apart from slightly improving precision (Table S3). Estimates from models without co-adjustment for the other OP metabolite sum were similar to the mutually adjusted estimates (Table S4).

## 4. Discussion

In this nested case-control study of prenatal ΣDMP and ΣDEP exposure at 17 weeks’ gestation and offspring ADHD, we observed no evidence of increased odds of ADHD in relation to increased exposure either overall or within strata of child sex. Despite adjusting for prenatal consumption of raw fruit and vegetables, higher exposure to ΣDMP and ΣDEP metabolites tended to be associated with slightly reduced odds of ADHD, however the confidence intervals were wide and included the null.

Although the bulk of the literature on prenatal OP exposure and offspring ADHD is mixed, the results from our study are consistent with five previous studies that did not observe associations with ADHD or ADHD-like behaviors [34,36,37,40,70]. Similar to our findings, a study of the Generation R cohort by van den Dries (2019) et al., found that higher prenatal ΣDEP concentrations were linked with fewer ADHD traits at 3, 6, and 10 years, as measured by the Child Behavior Checklist [37]. Although both we and van den Dries et al. attempted to account for the potential beneficial role of a healthy diet in statistical models, it is likely that both of our studies were impacted by residual negative confounding by a healthy diet, as diets high in fruits and vegetables are associated with numerous health benefits [71]. Dalsager (2019) et al. also found no association of the chlorpyrifos-specific biomarker 3,5,6-trichloro-2-pyridinol with ADHD symptoms in the preschool period [36].

Conversely, other studies have found associations of prenatal OP exposure with offspring ADHD symptoms and executive dysfunction [33,35,39,41]. In particular, we draw attention to our recent investigation of prenatal OP exposure on preschool executive functions in which we found consistent negative impacts on both parent- and teacher-rated executive functions across a range of domains in the MoBa cohort [41]. However, it is important to note that in exploratory analyses of this population, adverse impacts on executive functions were much stronger among children without preschool ADHD, suggesting that other pathways, including potentially heritable pathways, may be more important in the development of ADHD [10].

Although interactions with child sex have been reported for prenatal OP exposure and ADHD in some studies, we did not observe evidence of multiplicative EMM in our study. This finding is consistent with four other studies on prenatal OP exposure and ADHD or ADHD-like symptoms that explored EMM by child sex [36,37,38,39,40]. However, findings in our study are contrary to a study by Marks (2010) et al. that found significant associations between prenatal DAPs and most measures of ADHD and ADHD-like symptoms among boys only on both the additive and multiplicative scales [33]. Additionally, Fortenberry (2014) et al. also observed EMM, finding a significant association between prenatal chlorpyrifos exposure and ADHD-related outcomes among girls only, when assessing EMM on the additive scale (multiplicative EMM was not assessed) [34]. Although our study found no evidence of EMM, our study’s reliance on a clinical diagnosis of ADHD may undermine power to assess EMM as girls were less-often diagnosed (only 28% of the cases) [72].

A reason for discrepancies in the literature may be the challenges inherent in measurement of ADHD or ADHD-like symptoms, which may be magnified in the earliest age groups. The majority of prenatal OP-ADHD studies focused on symptoms in the preschool period [33,35,36,37,38,40], with only 3 other studies evaluating ADHD symptoms after the age of 5 years [34,37,39]. ADHD symptoms in the preschool period are particularly difficult to appropriately capture, because many symptoms of ADHD, like hyperactivity, are considered normative for this age group [73]. Our study is intended to be representative of ADHD cases in Norway, therefore, it is likely that few ADHD cases in this study received an ADHD diagnosis before the age of 6 [49]. Additionally, while our study is benefited by having a clinical diagnosis, clinical diagnosis of ADHD is challenging because diagnostic criteria are based on observation of external behaviors, and thus clinical variability and experience play a role in diagnosis at the provider level [74].

The OP-ADHD literature is also challenged by numerous study-specific differences regarding the nature of exposure to OP parent compounds in the individual populations, which are difficult to differentiate based on non-specific dialkylphosphate biomarkers. Half of the studies on prenatal OP exposure and ADHD were conducted in the US and more than a quarter of agricultural pesticides used in the US are currently banned in the EU, including many OPs [15,43,75]. Because the most common biomarkers of OPs used are non-specific DAPs, distinguishing between specific OPs with varying levels of toxicity is impossible. As such, differences in associations across studies may be in part due to a different compilation of parent compound exposures in the underlying communities. In addition, a number of studies have been conducted in higher-dose communities with substantial direct exposure to parent compounds through either agricultural or indoor pest applications [44,76,77,78]. Ye et al. (2009) directly compared DAP concentrations in MoBa, the Generation R Study, and NHANES, finding that MoBa concentrations were considerably lower than Generation R, but somewhat higher than NHANES, which the authors attributed to differences in dietary exposure across these regions [44]. However, dietary exposure to OPs as measured by urinary DAPs is susceptible to misclassification, as it is impossible to distinguish between exposure to the potentially toxic parent compound and the non-toxic metabolites themselves [79]. OPs on fruits and vegetables are known to biodegrade quickly [80] and the majority of food in Norway is imported [43,44,45,46]. Therefore, the DAP concentrations seen in our study may be reflective of a larger proportion of non-toxic OP metabolites rather than direct OP exposure.

Our study has many strengths. This was an efficient nested case-control study within a large and representative population-based cohort that contained an extensive amount of questionnaire data covering important covariates, such as maternal ADHD symptoms and prenatal dietary intake of fruits and vegetables. As a result, we were able to account for important confounding pathways that have been rarely addressed in prior studies. Maternal ADHD has the potential to confound the association between prenatal OP exposure and offspring ADHD through socioeconomic and behavioral pathways [65,66,67,68,81,82,83]. Fruit and vegetable consumption may be an important source of negative confounding due to the beneficial health impacts of a diet rich in fruit and vegetables [71]. Our study is the first studies to utilize clinically diagnosed ADHD. Most previous studies on prenatal OP exposure and offspring ADHD have relied on maternally reported symptoms of ADHD-like behaviors, which may be variably accurate. Diagnosed cases are more likely to select for clinically significant and impairing symptoms of ADHD, and are more likely to employ diagnostic standards, improving the accuracy of ADHD classification.

Although there are many strengths of our study, there are also some limitations. Because MoBa only collected one urine sample during pregnancy, we can only assess exposure at ~17 weeks’ gestation. OPs are quickly metabolized with estimated half-lives between a few hours to several days [56] and have been found to have low reliability among pregnant individuals [84]. Therefore, results may not be generalizable to other periods in pregnancy or post-natal exposure. Furthermore, although the second trimester is thought to be a particularly susceptible window of exposure for neurodevelopment because of rapid brain growth and development, the null results observed in this study could be due to OPs having a different window of susceptibility such as the first or third trimester of pregnancy or during the postnatal period [12,13]. However, van den Dries et al. reported similar results having three urine specimens over the course of pregnancy [37]. Additionally, while utilizing a clinical diagnosis of ADHD substantially improves accuracy over parentally reported symptoms, a recent study investigating the clinical basis of ADHD NPR registrations reported that only 50% of the sample examined had adequately documented the clinical evaluation supporting the diagnosis [85]. While these insufficiently documented cases may yet be true ADHD cases, the lack of appropriate documentation suggests the possibility of misclassification, even in a clinically assessed sample. Also, linkage with the NPR was conducted at a single point in time, which results in earlier birth years having more opportunity for diagnosis. To address this, we adjusted for birth year in our final model to account for any temporal factors that may be related to outcome ascertainment or exposure. Apart from clinical accuracy, other population-level selection factors may impact clinical ascertainment. For example, girls represent a minority of the NPR ADHD cases identified in this study (28%), which may in part be due to lower clinical referrals for ADHD evaluation in girls as a result of their lower prevalence of externalizing symptoms [72]. While under-identification of ADHD cases would not bias our primary results, it would reduce our power to identify EMM by sex [86]. Therefore, null results regarding sex-specific effects may be indicative of lack of power rather than lack of an association. Furthermore, this study used an ICD-10 classification of ADHD which differs from DSM diagnostic criteria as it does not recognize inattentive-only ADHD cases [50,87]. As such, care should be made in cross-study comparisons using different diagnostic criteria. Similarly, ADHD is a heterogenous disorder that can be classified into different subtypes such as hyperactive-only ADHD or hyperactive and inattentive ADHD. This study was unable to differentiate between ADHD subtypes; however, future studies may want to consider potential modification by ADHD subtype as the underlying biological mechanisms between OPs and ADHD could be subtype specific.

## 5. Conclusions

In summary, despite a robust body of evidence linking prenatal OP exposure with adverse neurodevelopmental impacts [31,32] including deficits in executive function [26,27], we did not observe an association between prenatal ΣDEP or ΣDMP exposure at 17 weeks’ gestation and increased odds of clinically diagnosed ADHD in offspring, even after accounting for fruit and vegetable intake and maternal ADHD symptoms. Although confidence intervals were wide (particularly among girls) and included the null, our results are in accordance with other recent studies that similarly found no association with ADHD [34,36,37,40,70]. OPs have short half-lives, and DAPs as biomarkers of exposure suffer from limitations which have been previously described [56,84]. In addition, ADHD is a complex disorder with strong heritability and there are few modifiable risk factors that have been strongly linked with its occurrence, however more research is urgently needed in this area in order to reduce the prevalence of this debilitating disorder.

## Figures and Tables

**Table 1:** Characteristics of a nested case-control study of attention-deficit/hyperactivity disorder (ADHD) in the Norwegian Mother, Father, and Child Cohort Study, birth years 2003–2008.

Characteristic	ADHD Cases	Representative Controls
	*Mean* (*SD*) or N (%)	*Mean* (*SD*) or N (%)
Total N	297	552
Maternal age at delivery (years)	*29.2* (*5.1*)	*30.9* (*4.2*)
Missing (N)	2	2
Child sex at birth		
Male	214 (72.3)	274 (49.6)
Female	82 (27.7)	278 (50.4)
Missing (N)	1	0
Maternal education		
Less than a 4-year college degree	160 (61.8)	123 (23.3)
4-year college degree	74 (28.6)	238 (45.0)
More than a 4-year college degree	25 (9.7)	168 (31.8)
Missing (N)	38	23
Experienced financial difficulty in the past 12 months		
Yes	102 (38.1)	73 (13.3)
No	166 (61.9)	477 (86.7)
Missing (N)	29	2
Marital status		
Single	18 (6.7)	14 (2.6)
Co-inhabiting	144 (53.5)	245 (44.8)
Married	107 (39.8)	288 (52.7)
Missing (N)	28	5
Parity		
Nulliparous	154 (52.2)	281 (51.1)
Parous	141 (47.8)	269 (48.9)
Missing (N)	2	2
Maternal ADHD symptoms *		
Yes	11 (8.0)	21 (3.9)
No	126 (92.0)	520 (96.1)
Missing (N)	160	11
Reported lifetime history of depression		
Yes	96 (36.5)	115 (21.1)
No	167 (63.5)	429 (78.9)
Missing (N)	34	8
Any smoking during pregnancy		
Yes	94 (34.8)	77 (14.1)
No	176 (65.2)	469 (85.9)
Missing (N)	27	6
Any alcohol use during pregnancy		
Yes	26 (10.8)	66 (13.0)
No	214 (89.2)	442 (87.0)
Missing (N)	57	44
Raw-vegetable consumption (servings/day)	*0.47* (*0.39*)	*0.60* (*0.48*)
Missing (N)	48	12
Raw fruit consumption (servings/day)	*1.90* (*1.45*)	*2.13* (*1.26*)
Missing (N)	44	13
Organic vegetable consumption		
Seldom/Never	167 (66.5)	328 (61.2)
Sometimes/Often/Usually	84 (33.5)	208 (38.8)
Missing (N)	46	16
Organic fruit consumption		
Seldom/Never	182 (72.2)	361 (67.5)
Sometimes/Often/Usually	70 (27.8)	174 (32.5)
Missing (N)	45	17
Total fish consumption (grams/day)	*26.9* (*20.4*)	*27.5* (*19.0*)
Missing (N)	41	7
Maternal exposure to pesticides during the past 6 months ^†^		
Yes	8 (3.2)	25 (4.9)
No	241 (96.8)	483 (95.1)
Missing (N)	48	44
Paternal exposure to pesticides during the past 6 months ^†^		
Yes	26 (12.3)	56 (11.9)
No	185 (87.7)	414 (88.1)
Missing (N)	86	82
Resided on a farm or detached home during pregnancy		
Yes	119 (45.8)	220 (41.7)
No	141 (54.2)	308 (58.3)
Missing (N)	37	24
Year of birth		
2003–2004	131 (44.1)	55 (10.0)
2005	87 (29.3)	130 (23.6)
2006	44 (14.8)	194 (35.1)
2007–2008	35 (11.8)	173 (31.3)
Missing (N)	0	0
Season of urine collection		
Fall	56 (18.9)	121 (21.9)
Winter	67 (22.6)	155 (28.1)
Spring	81 (27.3)	140 (25.4)
Summer	93 (31.3)	136 (24.6)
Missing (N)	0	0

Note: SD, standard deviation; N, frequency. Note: Percentages may not add to 100% due to rounding. * Based on the Adult ADHD Self-Report Scale (SRS). ^†^ Refers to any exposure (yes/no) to weed killers, insecticides, and fungicides during the six months prior to the questionnaire distributed at 17 weeks’ gestation.

**Table 2:** Specific-gravity-corrected organophosphorus metabolite distribution at 17 weeks’ gestation in a nested case-control study of attention-deficit/hyperactivity (ADHD) in the Norwegian Mother, Father, and Child Cohort Study (MoBa), birth years 2003–2008 (N = 849).

Exposure	Geometric Mean (SD) *	Min	25%	50%	75%	Max
DMP (nmol/L)						
ADHD cases (N = 297)	23.0 (3.86)	1.01	6.54	27.1	66.8	617
Representative MoBa controls (N = 552)	31.3 (3.73)	0.03	10.7	36.3	80.0	1057
DMTP (nmol/L)						
ADHD cases (N = 297)	16.0 (4.44)	0.20	5.87	14.0	40.9	1015
Representative MoBa controls (N = 552)	26.2 (3.89)	0.01	10.3	22.7	59.4	1291
DMDTP (nmol/L)						
ADHD cases (N = 297)	2.58 (3.15)	0.24	1.34	2.07	4.13	180
Representative MoBa controls (N = 552)	3.32 (3.62)	0.01	1.43	2.76	5.90	356
ΣDMP (nmol/L)						
ADHD cases (N = 297)	49.4 (3.41)	5.88	17.9	45.6	117	1412
Representative MoBa controls (N = 552)	71.2 (3.29)	0.05	30.9	64.6	155	1979
DEP (nmol/L)						
ADHD cases (N = 297)	11.7 (2.31)	0.46	6.59	11.3	20.8	162
Representative MoBa controls (N = 552)	14.5 (2.39)	0.01	8.54	14.2	24.6	124
DETP (nmol/L)						
ADHD cases (N = 297)	2.55 (3.95)	0.09	0.88	2.25	6.10	206
Representative MoBa controls (N = 552)	4.32 (4.15)	0.01	1.62	4.16	10.5	541
ΣDEP (nmol/L)						
ADHD cases (N = 297)	15.8 (2.42)	0.76	8.39	13.9	30.6	229
Representative MoBa controls (N = 552)	21.0 (2.58)	0.02	11.5	19.7	37.1	581

Note: Concentrations were expressed to three significant digits, except for the maximum (max) value. Representative MoBa controls were randomly selected from the eligible population to represent the exposure distribution in the study base. * SD refers to the geometric standard deviation. Min, minimum; max, maximum; nmol/L, nanomole per liter; DMP, dimethyl phosphate; DMTP, dimethyl thiophosphate; DMDTP, dimethyl dithiophosphate; ΣDMP, the molar sum of the dimethyl phosphates; DEP, diethyl phosphate; DETP, diethyl thiophosphate; ΣDEP, the molar sum of the diethyl phosphates. Values below the limit of detection were imputed from a log-normal distribution truncated at the limit of detection. All values were standardized to the geometric mean of specific gravity.

**Table 3:** Estimated overall and sex-specific odds of having a child with attention-deficit/hyperactivity disorder (ADHD) per tertile of organophosphorus pesticide metabolite concentration at 17 weeks’ gestation, adjusted for covariates, in a nested case-control study of ADHD in Norwegian Mother, Father, and Child Cohort Study, birth years 2003–2008.

Exposure	Combined		Boys *		Girls *	
	Cases/Controls	OR (95% CI)	Cases/Controls	OR (95% CI)	Cases/Controls	OR (95% CI)
∑DMP						
Tertile 1 (<34.5 nmol/L)	126/157	ref	88/70	ref	37/87	ref
Tertile 2 (34.6 to 102.6 nmol/L)	87/196	0.77 (0.49, 1.21)	67/103	0.70 (0.40, 1.23)	20/93	0.70 (0.30, 1.61)
Tertile 3 (>102.6 nmol/L)	84/199	0.63 (0.38, 1.04)	59/101	0.55 (0.29, 1.04)	25/98	0.65 (0.27, 1.59)
∑DEP						
Tertile 1 (<12.4 nmol/L)	129/154	ref	94/70	ref	34/84	ref
Tertile 2 (12.5 to 26.3 nmol/L)	88/195	0.86 (0.54, 1.36)	62/100	0.72 (0.41, 1.29)	26/95	1.41 (0.61, 3.28)
Tertile 3 (>26.3 nmol/L)	80/203	0.83 (0.50, 1.39)	58/104	0.80 (0.43, 1.52)	22/99	0.86 (0.34, 2.15)

Note: OR, odds ratio; CI, confidence interval; ref, reference; ΣDMP is the molar sum of the dimethyl phosphates; ΣDEP is the molar sum of the diethyl phosphates; nmol/L, nanomole per liter. Models are adjusted for season, birth year, maternal education, vegetable intake, fruit intake, maternal self-reported ADHD, financial status, other OP molar sum, and sex. Stratum-specific estimates are derived from models that additionally include interaction terms for each included variable using an augmented product term approach to assess effect measure modification (EMM) by sex on the multiplicative scale. * *p*-values for EMM were all ≥0.20.

## Data Availability

All inquiries related to obtaining data from the Norwegian Mother, Father and Child Cohort Study (MoBa) should be directed to the MoBa executive officer at the Norwegian Institute of Public Health (Mobaadmin@fhi.no). Analytic code used for the present analysis may be obtained from the corresponding author.

## Supplementary Materials

The following supporting information can be downloaded at: https://www.mdpi.com/article/10.3390/ijerph192416851/s1, Table S1. Non-specific-gravity standardized organophosphorus metabolite distribution at 17 weeks’ gestation in a nested case-control study of attention-deficit/hyperactivity (ADHD) in the Norwegian Mother, Father, and Child Cohort Study (MoBa), birth years 2003–2008; Table S2. Spearman correlations between molar sums of organophosphorus pesticide metabolites at 17 weeks’ gestation in a nested case control study of attention-deficit/hyperactivity disorder (ADHD) in the Norwegian Mother, Father, and Child Cohort Study (MoBa), birth years 2003–2008; Table S3. Sensitivity analysis evaluating the effects of imputing covariate data in the assessment of organophosphorus pesticide metabolite concentrations at 17 weeks’ gestation and attention-deficit/hyperactivity disorder in a nested case-control study of ADHD in Norwegian Mother, Father, and Child Cohort, birth years 2003–2008; Table S4. Sensitivity analysis for the mutual adjustment of the other organophosphorus pesticide metabolite molar sum in the evaluation of organophosphorus pesticide exposure at 17 weeks’ gestation and child attention-deficit/hyperactivity disorder in a nested case-control study within the Norwegian Mother, Father, and Child Cohort, birth years 2003–2008.
