# Supplementary material for: Organophosphorus Pesticide Exposure at 17 Weeks’ Gestation and Odds of Offspring Attention-Deficit/Hyperactivity Disorder Diagnosis in the Norwegian Mother, Father, and Child Cohort Study
Source: Int J Environ Res Public Health. 2022 Dec 15;19(24):16851. doi: 10.3390/ijerph192416851 (PMC9778918; doi:10.3390/ijerph192416851)
Supplement: Supplementary file 1 [file ijerph-19-16851-s001.zip › ijerph-1995666-supplementary.pdf]

Supplemental Material for Organophosphorus Pesticide Exposure at 17 Weeks' Gestation and Odds of Attention-Deficit/Hyperactivity Disorder Diagnosis in the Norwegian Mother, Father, and Child Cohort Study

Authors: Amber M. Hall, Jake E. Thistle, Cherrel K. Manley, Kyle R. Roell, Amanda M. Ramos, Gro D. Villanger, Ted Reichborn-Kjennerud, Pål Zeiner, Enrique Cequier, Amrit K. Sakhi, Cathrine Thomsen, Heidi Aase, Stephanie M. Engel

Table of Contents

Table S1. Non-specific-gravity standardized organophosphorus metabolite distribution at 17 weeks' gestation in a nested case-control study of attention-deficit/hyperactivity (ADHD) in the Norwegian Mother, Father, and Child Cohort Study (MoBa), birth years 2003-2008 2

Table S2. Spearman correlations between molar sums of organophosphorus pesticide metabolites at 17 weeks' gestation in a nested case control study of attention-deficit/hyperactivity disorder (ADHD) in the Norwegian Mother, Father, and Child Cohort Study (MoBa), birth years 2003-2008........ 3

Table S3. Sensitivity analysis evaluating the effects of imputing covariate data in the assessment of organophosphorus pesticide metabolite concentrations at 17 weeks' gestation and attention-deficit/hyperactivity disorder in a nested case-control study of ADHD in Norwegian Mother, Father, and Child Cohort, birth years 2003-2008........ 4

Table S4. Sensitivity analysis for the mutual adjustment of the other organophosphorus pesticide metabolite molar sum in the evaluation of organophosphorus pesticide exposure at 17 weeks' gestation and child attention-deficit/hyperactivity disorder in a nested case-control study within the Norwegian Mother, Father, and Child Cohort, birth years 2003-2008 5

Table S1. Non-specific-gravity standardized organophosphorus metabolite distribution at 17 weeks' gestation in a nested case-control study of attention-deficit/hyperactivity (ADHD) in the Norwegian Mother, Father, and Child Cohort Study (MoBa), birth years 2003-2008

Exposure	Geometric mean	Geometric SD	Min	25%	50%	75%	Max	%>LOD	%>LOQ
DMP (ng/mL)									
ADHD cases (N=297)	6.09	2.80	<LOD	<LOD	2.53	7.14	84.9	61.6%	47.8%
Representative MoBa controls (N=552)	6.51	2.81	<LOD	<LOD	3.19	10.1	168	68.5%	51.6%
DMTP (ng/mL)									
ADHD cases (N=297)	2.04	4.71	<LOD	0.73	1.95	4.91	124	99.7%	85.2%
Representative MoBa controls (N=552)	3.01	4.20	0.09	1.03	2.52	7.99	221	100.0%	92.9%
DMDTP (ng/mL)									
ADHD cases (N=297)	1.52	3.81	<LOD	<LOD	<LOD	<LOD	30.9	22.6%	11.5%
Representative MoBa controls (N=552)	1.58	3.38	<LOD	<LOD	<LOD	0.60	83.9	32.3%	16.7%
DEP (ng/mL)									
ADHD cases (N=297)	1.66	2.50	<LOD	0.84	1.42	3.11	32.8	98.0%	63.3%
Representative MoBa controls (N=552)	1.84	2.59	<LOD	0.88	1.68	3.46	25.9	99.1%	67.2%
DETP (ng/mL)									
ADHD cases (N=297)	0.92	3.20	<LOD	<LOD	0.23	0.99	26.3	57.6%	34.7%
Representative MoBa controls (N=552)	1.11	3.36	<LOD	<LOD	0.52	1.58	131	71.7%	47.1%
DEDTP (ng/mL)									
ADHD cases (N=297)	0.18	1.87	<LOD	<LOD	<LOD	<LOD	0.69	2.4%	0.3%
Representative MoBa controls (N=552)	0.16	1.25	<LOD	<LOD	<LOD	<LOD	0.23	2.4%	0.0%

Note: Concentrations were expressed to three significant digits.
Representative MoBa controls were randomly selected from the eligible population to represent the exposure distribution in the study base.
SD, standard deviation; min, minimum; max, maximum; ng/mL, nanogram per milliliter; DMP, dimethyl phosphate; DMTP, dimethyl thiophosphate; DMDTP, dimethyl dithiophosphate; ∑DMP, the molar sum of the dimethyl phosphates; DEP, diethyl phosphate; DETP, diethyl thiophosphate; ∑DEP, the molar sum of the diethyl phosphates.
Values below the limit of detection were imputed from a log-normal distribution truncated at the limit of detection.
All values were standardized to the geometric mean of specific gravity.

Table S2. Spearman correlations between molar sums of organophosphorus pesticide metabolites at 17 weeks' gestation in a nested case control study of attention-deficit/hyperactivity disorder (ADHD) in the Norwegian Mother, Father, and Child Cohort Study (MoBa), birth years 2003-2008

	∑DMP	
	ADHD cases (N=297)	MoBa Controls (N=552)
∑DEP		
ADHD cases (N=297)	0.489	-
MoBa Controls (N=552)	-	0.524

Values reported are Spearman correlation coefficients.
∑DMP is the molar sum of the dimethyl phosphates; ∑DEP is the molar sum of the diethyl phosphates.

Table S3. Sensitivity analysis evaluating the effects of imputing covariate data in the assessment of organophosphorus pesticide metabolite concentrations at 17 weeks' gestation and attention-deficit/hyperactivity disorder in a nested case-control study of ADHD in Norwegian Mother, Father, and Child Cohort, birth years 2003-2008

Exposure	Crude[a]		Not-imputed covariate data[b]		Imputed covariate data[b]	
	Cases/Controls	OR (95% CI)	Cases/Controls	OR (95% CI)	Cases/Controls	OR (95% CI)
∑DMP						
Tertile 1 (<34.5 nmol/L)	126/157	ref	106/154	ref	126/157	ref
Tertile 2 (34.6 to 102.6 nmol/L)	87/196	0.55 (0.39, 0.78)	78/191	0.84 (0.45, 1.55)	87/196	0.77 (0.49, 1.21)
Tertile 3 (>102.6 nmol/L)	84/199	0.53 (0.37, 0.74)	75/184	0.89 (0.46, 1.75)	84/199	0.63 (0.38, 1.04)
∑DEP						
Tertile 1 (<12.4 nmol/L)	129/154	ref	111/111	ref	129/154	ref
Tertile 2 (12.5 to 26.3 nmol/L)	88/195	0.54 (0.38, 0.76)	189/81	0.75 (0.39, 1.41)	88/195	0.86 (0.54, 1.36)
Tertile 3 (>26.3 nmol/L)	80/203	0.47 (0.33, 0.67)	193/67	0.68 (0.35, 1.34)	80/203	0.83 (0.50, 1.39)

Note: OR, odds ratio; CI, confidence interval; ref, reference; ∑DMP is the molar sum of the dimethyl phosphates; ∑DEP is the molar sum of the diethyl phosphates; nmol/L, nanomole per liter.

[a]Crude model refers to an unadjusted model assessing only the DMP or DEP molar sum respectively .

[b]Model is adjusted for season, birth year, maternal education, vegetable intake, fruit intake, maternal self-reported ADHD, financial status, other OP molar sum, and sex.

Table S4. Sensitivity analysis for the mutual adjustment of the other organophosphorus pesticide metabolite molar sum in the evaluation of organophosphorus pesticide exposure at 17 weeks' gestation and child attention-deficit/hyperactivity disorder in a nested case-control study within the Norwegian Mother, Father, and Child Cohort, birth years 2003-2008

Exposure	Mutually adjusted for the other OP metabolite OR (95% CI)	Not mutually adjusted for the other OP metabolite OR (95% CI)
∑DMP		
Tertile 1 (<34.5 nmol/L)	ref	ref
Tertile 2 (34.6 to 102.6 nmol/L)	0.77 (0.49, 1.21)	0.73 (0.47, 1.14)
Tertile 3 (>102.6 nmol/L)	0.63 (0.38, 1.04)	0.58 (0.37, 0.91)
∑DEP		
Tertile 1 (<12.4 nmol/mL)	ref	ref
Tertile 2 (12.5 to 26.3 nmol/L)	0.86 (0.54, 1.36)	0.76 (0.49, 1.19)
Tertile 3 (>26.3 nmol/L)	0.83 (0.50, 1.39)	0.68 (0.43, 1.07)

Note: OR, odds ratio; CI, confidence interval; ref, reference; ∑DMP is the molar sum of the dimethyl phosphates; ∑DEP is the molar sum of the diethyl phosphates; nmol/mL, nanomole per liter.

Model is adjusted for season, birth year, maternal education, vegetable intake, fruit intake, maternal self-reported ADHD, financial status, other OP molar sum, and sex.
